# Dr. David H. Tucker’s Case of Lumbar Abscess, with Remarks

**Published:** 1842-07-16

**Authors:** David H. Tucker

**Affiliations:** Philadelphia


### *A Case of Lumbar Abscess, with Remarks.* By DAVID H. TUCKER, M. D., of Philadelphia.

William Fleming is 34 years of age, and a carpenter by trade. Some time during last autumn he suffered greatly from dysentery. About six weeks after his recovery, he, for the first time, felt a severe pain in his back, and about Christmas a pain, of an excruciating character, attacked the right femoral articulation, extending itself down the thigh.

I saw him, for the first time, about the 15th of February last. He was tall and thin, but had enjoyed excellent health previous to his attack of dysentery. He has no cough; his bowels are now constipated. There is no appearance of a tumour in his abdomen; he makes his water freely; urine slightly sedimentous, but contains neither pus nor blood; his appetite is bad; he complains greatly of pain in his back and right femoral articulation; his thigh and leg are flexed, so that it is impossible to extend them; the limb of the opposite side is not affected. On examining the spine I found no deviation in the vertebræ, but on pressing at a point, to the right of the two last lumbar vertebræ, he experienced a great deal of pain, and here I thought I discovered a slight swelling. The coxo-femoral articulation is neither swollen nor red, nor is the pain increased by pressure along the nerve. In order to relieve the pain, I prescribed opiates internally, and hot hop fomentations over the seat of pain.

Some days after this I saw the patient in consultation with Dr. Page, and we both came to the conclusion that there existed an abscess to the right of the lumbar vertebræ. The tumour and fluctuation were not very apparent. We continued the internal use of the opium, and applied a blister over the tumour, directing it to be kept open by frequent dressing with savine ointment.

Four days after this I found the tumour had increased, and the fluctuation was so apparent that I determined to open it. The opening was made, and an enormous quantity of pus was evacuated. It consisted of a mixture of pus and blood, and had a very offensive odour. The quantity discharged at this time amounted to about a teacupful. On probing, I could discover no denuded bones. The extent of the abscess was as follows: Depth, *forward*, towards the anterior walls of the abdomen, measures two inches; *upward*, about *one* inch; and downward, towards the sacro iliac junction, the depth was more than *four* inches. Treatment consisted in the exhibition of a solution of quinine and aromatic sulphuric acid. Poultices over the opening.

Feb. 24th. Discharge from the abscess has been very great; pain less; profuse sweats. A second tumour has appeared in the groin, and a third to the right of the sacro iliac junction. The cavity of this latter appears to communicate with the abscess, which has been opened.

Feb. 26th. Discharge from the abscess has diminished; tumour in the groin is soft and fluctuating; sweating still continues; patient has a good appetite, and says he is no weaker, and has less pain.

Feb. 28th. Discharge from abscess has been very great, but the matter is now of a cream colour, and not so offensive. Both of the other tumours were opened, and discharged a great deal. The one in the groin gave exit to an enormous quantity of gray matter, very offensive, and mixed up with a large quantity of gas. Patient suffers greatly; constipation. Treatment continued, with the addition of an aperient.

March 2d. The abscesses continue to discharge a great deal; pain less; sleeps well; bowels open; no appetite. Treatment same as before, except the addition of brandy punch.

March 6th. Pain very great; abscesses discharge as usual; appetite improved; sweating continues very profuse; pulse weak. Treatment continued.

March 20th. Patient has been doing quite well, and although the discharge is still very great, his general health is better. Appetite tolerable; sweating less profuse; more strength.

April 20th. The patient's condition has changed very little during the last month, but on the 25th of April he had a profuse discharge of blood and pus from the bowels, and while this continued the discharge from abscesses was very much lessened. Diarrhœa has debilitated him greatly; night sweats very profuse; pulse feeble; appetite bad. Treatment continued. Large doses of laudanum prescribed to check the diarrhœa.

May 20th. The diarrhœa has continued, though from time to time it has diminished more or less. The discharge from the abscess varies as the diarrhœa is *more* or *less* violent. The strength of the patient is much less; no appetite; pulse very feeble; hiccups constant; tongue very red around the edges, the centre is covered with a white fur. Brandy was prescribed to relieve the hiccup, otherwise the treatment was the same.

May 23d. Diarrhœa continues; the abscesses discharge very little; those in the back are nearly dried up; pain over the sacrum very severe; hiccup gone, otherwise the patient's state is not changed. Treatment the same. Large doses of tincture of opium in the form of enema.

May 25th. Diarrhœa rather better, but the patient is sinking rapidly; no appetite; pulse feeble; hands and feet cold; abscesses discharge but little. Treatment continued.

Died on evening of the 26th.

### *Autopsy thirty-six hours after Death.*

The abscess extended up along the right side of the spinal column as far as the dorsal vertebræ. The bones of the sacrum, ilium, and the two last lumbar vertebræ, were carious; and, under the periosteum of these last, there was a good deal of pus. The abscess contained some pus of a gray colour. The walls of the cavity were of a dark colour and very soft, and lined with a false membrane. The psoas and iliacus muscles were of a dark colour, and had lost their consistence; they contained masses of tuberculous matter, about the size of a pea, scattered throughout their tissue. Leading from the cavity of the abscess were several sinuses, one passed backwards to the lumbar region, the other passed down in front of the psoas muscle and opened in the groin; a third passed down across the sacrum, opening into the rectum, about two inches above the *anus.* Nearly four inches of the rectum was in a gangrenous state. At the caput coli there were strong adhesions, binding the intestines to the walls of the abdomen. The gut was at this point also perforated, and about two inches of it was gangrenous. The intestines were healthy in every other point, except a few old adhesions.

*Thorax.*—The lungs were perfectly healthy. Old pleuritic adhesions existed on the right side. The pericardium was filled with water. Heart was small but healthy throughout, except that there existed the remains of a false membrane on its external surface. No adhesions between the pericardium and heart.

The liver, spleen, kidneys and stomach, were all healthy. The brain was not examined.

### *Remarks.*

Authors have divided abscesses into acute and chronic; the latter is of two kinds. 1st. Where the collection of matter shows itself at the point where the pus is secreted. 2d. Where the tumour appears at a point distant from that at which the pus is formed, or *an abscess by congestion.* The case reported is of the last class; and the pus, formed in the cellular tissue of the loins, behind the peritoneum, passes along the psoas muscle and points at the groin, or, finding its way into the pelvis, shows itself at some part of the perineum. The abscesses are strictly chronic, and in a large majority of cases are preceded by a disease of the vertebræ. Dr. Physick never saw a case, in America, unconnected with caries of the vertebræ. The thickness of the posterior walls of the abdomen—the vertical position of the body—the pressure of the intestines—and the great mass of loose cellular tissue about the vertebræ and *basin*, *all* favour the flow of pus towards the groin and perineum.

Cayol and Gooch have each reported a case in which the abscess opened into the lungs. And about a year since I had a case of this disease, in which the patient stated that previously a lumbar abscess had opened into the lungs.

Wedemeyer met with a case where the abscess opened into the colon; and in the case reported, there were two openings in the intestines, one in the colon and the other in the rectum. These abscesses, generally, are a long time in forming, and their duration is very variable. Their cure is possible, but very rare. They may be confounded with aneurisms, hernias, buboes, and sometimes with acute abscess in the iliac regions. In this case, where there was no reason to suspect a disease of the vertebræ, we at first suspected either a neuralgia or a disease of the kidneys. Was the inflammation of the bowels the starting point of the disease in this case? or was the disease of the vertebræ the cause?

As to the propriety of opening these abscesses, Roux and Béclard say, 1st. We should never permit them to open spontaneously, as the opening will scarcely ever close. 2d. We should defer opening to the latest moment—taking care not to permit the walls of the tumour to become *too thin*. 3d. We should not permit the air to enter the cavity of the abscess. S. Cooper says, in his Dictionary: "But still, the principle (of opening all chronic abscesses while small) is praiseworthy, and should urge us to open the tumour as soon as the fluctuation of the matter is distinct, and the nature of the case is evident."

